# The effect of clinical decision support systems on clinical outcomes in acute kidney injury: a systematic review and meta-analysis of randomized controlled trials

**DOI:** 10.1080/0886022X.2024.2400552

**Published:** 2024-09-09

**Authors:** Obieda Altobaishat, Mohamed Abouzid, Ahmed Mazen Amin, Abdallah Bani-Salameh, Mohammad Tanashat, Omar Abdullah Bataineh, Mustafa Turkmani, Mohamed Abuelazm, Muner M. B. Mohamed

**Affiliations:** a Faculty of Medicine, Jordan University of Science and Technology, Irbid, Jordan; b Department of Physical Pharmacy and Pharmacokinetics, Faculty of Pharmacy, Poznan University of Medical Sciences, Poznan, Poland; c Doctoral School, Poznan University of Medical Sciences, Poznan, Poland; d Faculty of Medicine, Mansoura University, Mansoura, Egypt; e Faculty of Medicine, Yarmouk University, Irbid, Jordan; f Faculty of Medicine, Michigan State University, East Lansing, MI, USA; g Department of Internal Medicine, McLaren Health Care, Oakland, MI, USA; h Faculty of Medicine, Tanta University, Tanta, Egypt; i Department of Nephrology, Ochsner Health System, New Orleans, LA, USA; j Ochsner Clinical School, The University of Queensland, Brisbane, Australia

**Keywords:** Acute kidney injury, care bundle, electronic alert, clinical decision support system

## Abstract

**Objectives:**

To determine whether clinical decision support systems (CDSS) for acute kidney injury (AKI) would enhance patient outcomes in terms of mortality, dialysis, and acute kidney damage progression.

**Methods:**

The systematic review and meta-analysis included the relevant randomized controlled trials (RCTs) retrieved from PubMed, EMBASE, Web of Science, Cochrane, and SCOPUS databases until 21st January 2024. The meta-analysis was done using (RevMan 5.4.1). PROSPERO ID: CRD42024517399.

**Results:**

Our meta-analysis included ten RCTs with 18,355 patients. There was no significant difference between CDSS and usual care in all-cause mortality (RR: 1.00 with 95% CI [0.93, 1.07], *p* = 0.91) and renal replacement therapy (RR: 1.11 with 95% CI [0.99, 1.24], *p* = 0.07). However, CDSS was significantly associated with a decreased incidence of hyperkalemia (RR: 0.27 with 95% CI [0.10, 0.73], *p* = 0.01) and increased eGFR change (MD: 1.97 with 95% CI [0.47, 3.48], *p* = 0.01).

**Conclusions:**

CDSS were not associated with clinical benefit in patients with AKI, with no effect on all-cause mortality or the need for renal replacement therapy. However, CDSS reduced the incidence of hyperkalemia and improved eGFR change in AKI patients.

## 1. Introduction

Acute kidney injury (AKI) is a prevalent grave complication that impacts hospital admissions [1]. AKI affects more than 50% of patients in the intensive care unit (ICU) and up to 18% of general inpatients [2]. Also, AKI has significant resource and financial ramifications since it raises the risk of in-hospital mortality, the onset of cardiovascular disease, and the development of chronic kidney disease [3]. Early identification and effective management of AKI are essential to promote renal recovery and avoid associated negative consequences [4].

Medication errors may be decreased significantly with the use of clinical decision support (CDS) in computerized provider order entry (CPOE) and electronic medical records (EMRs) [5,6]. Serum creatinine (SCr) variations are used by the automated algorithm that generates the electronic AKI alert to detect AKI early [7]. Clinicians can immediately act When they receive notification of an AKI episode [7]. Recently, automated alerts have become a powerful tool for influencing physician behavior [8]. Randomized controlled trials (RCTs) in the hospital setting have demonstrated the effectiveness of alerts in reducing medication interactions [9].

A recent compilation of research findings from multiple studies on clinical decision support systems (CDSS) found that while only 30% of studies positively impacted patient outcomes, 57% affected practitioner behavior [10]. Based on these considerations, this systematic review and meta-analysis of RCTs was conducted to determine whether an automated AKI alert would reduce AKI severity and improve clinical outcomes (mortality, hyperkalemia, progression of AKI, length of hospital stay, and others) in hospitalized patients. To the best of our knowledge, this study is the most up-to-date and thorough synthesis of the available data in this quickly developing field of study. The possible conclusions drawn from this review are expected to have significant implications. They may help close a considerable knowledge gap regarding the contribution of CDSS to AKI patients’ health and may also direct future medical procedures and treatment plans.

## 2. Methodology

### 2.1. Protocol registration

The Cochrane Handbook for Systematic Reviews and Meta-Analyses [11] and the PRISMA (Preferred Reporting Items for Systematic Reviews and Meta-Analyses) statement [12] guided the conduct of this systematic review and meta-analysis. The protocol for this review has been registered and published in PROSPERO with the following ID: CRD42024517399.

### 2.2. Data sources and search strategy

Up until 21st January 2024, O.A. and M.A.azm searched several databases, including PubMed (MEDLINE), EMBASE, Web of Science, SCOPUS, and the Cochrane Central Register of Controlled Trials (CENTRAL). No filters or search restrictions were applied. Table S1 contains more information about the search strategy, including the keywords, search terms, and the search results.

### 2.3. Eligibility criteria

A Population, Intervention, Comparison, and Outcomes (PICO) criterion was used to include RCTs: population (P): inpatient adults aged 18 years or older with AKI; intervention (I) CDSS for AKI alert and care bundle; control (C): usual care; and outcomes (O): primary outcomes: all-cause mortality and renal replacement therapy, secondary outcomes: hyperkalemia (serum potassium >5.5 mmol/L), estimated glomerular filtration rate (eGFR) change (eGFR at AKI minus the lowest eGFR within the time of randomization (from admission to the discharge), If the patient died, eGFR was considered to be equal to zero, re-hospitalization, creatinine change (serum creatinine between hospital admission and highest serum creatinine within time of randomization), number of patients received non-steroidal anti-inflammatory drugs (NSAIDs), aminoglycosides, fluids, angiotensin-converting enzyme inhibitors (ACEi) and angiotensin II receptor blockers (ARBs), contrast, AKI on discharge, nephrologist consultation, progression of AKI, renal recovery (Total; <1.2 times of baseline serum creatinine, partial; ≥1.2 times and 1.5 times of baseline serum creatinine), renal ultrasonography within 2 days, length of hospital stay (days), and hospital cost.

The exclusion criteria included studies that were conducted on animals, pilot projects, unpublished study protocols, different types of observational studies, such as cohorts, case-control, cross-sectional, case series, and case reports, single-arm clinical trials, *in vitro* experiments on tissues and cultures, book chapters, editorials, press articles, publications that only contained abstracts or posters, and studies published in languages other than English.

### 2.4. Study selection

The Covidence online software was used to conduct the review process. After removing any duplicate records, four reviewers independently completed them (M.T.ani, M.T.hat, O.B., and A.B.). Full-text screening was used to review the complete texts of the records that initially satisfied the eligibility requirements. Any disagreements were settled through conversations involving (O.A.).

### 2.5. Data extraction

Using a pre-made extraction sheet, four reviewers (M.T.ani, M.T.hat, O.B., and A.B.) separately extracted the following data: summary characteristics (study design, country, total participants, registry number, number of centers, AKI alert details, control, main inclusion criteria, follow-up duration, and primary outcome); baseline characteristics (number of participants in each group, age, gender, basal metabolic index, smoking, hospital admissions at intensive care unit (ICU), AKI grade, comorbidities (diabetes, hypertension, liver disease, chronic obstructive pulmonary disease (COPD), cerebrovascular disease, congestive heart failure, chronic kidney disease); efficacy and safety data (primary and secondary outcomes) as previously described. Any disagreements were resolved through discussion with the first author (O.A.).

### 2.6. Risk of bias and certainty of evidence

Using the Cochrane RoB2 tool, four reviewers (M.T.ani, M.T.hat, O.B., and A.B.) independently evaluated the quality of the studies included in the research [13]. The risk of bias arising from the randomization process, the risk of bias resulting from deviating from the intended intervention, the risk of bias resulting from missing outcome data, the risk of bias in measuring the outcome, and the risk of bias resulting from choosing the published results were among the domains that were assessed. If there were any conflicts, the reviewers discussed them and reached a consensus.

For the certainty of evidence, we followed the Grading of Recommendations Assessment, Development, and Evaluation (GRADE) recommendations [14,15], considering inconsistency, imprecision, indirectness, publication bias, and risk of bias. The evaluation was carried out for each outcome, and the decisions were justified and documented. Any discrepancies were settled through discussion.

### 2.7. Statistical analysis

RevMan v5.4 was used to conduct the statistical analysis. For continuous outcomes, we used the mean difference (MD) with a 95% confidence interval (CI), and for dichotomous outcomes, we used the risk ratio (RR). We performed both the Chi-square and I-square tests to evaluate heterogeneity, where the Chi-square test detects the presence of heterogeneity, and the I-square test evaluates its degree. We used the random-effects model when there was significant heterogeneity (*I*^2^ > 50%) and the common-effect model when heterogeneity was not significant (*I*^2^ < 50%). The I-square was interpreted as follows by the Cochrane Handbook (chapter nine): heterogeneity is moderate for 30–60%, substantial for 50–90%, and considerable for 75–100%. It is not significant for 0–40%. In case of significant heterogeneity, a leave-one-out sensitivity analysis was conducted to investigate the source of heterogeneity.

## 3. Results

### 3.1. Search results and study selection

**Figure 1.**
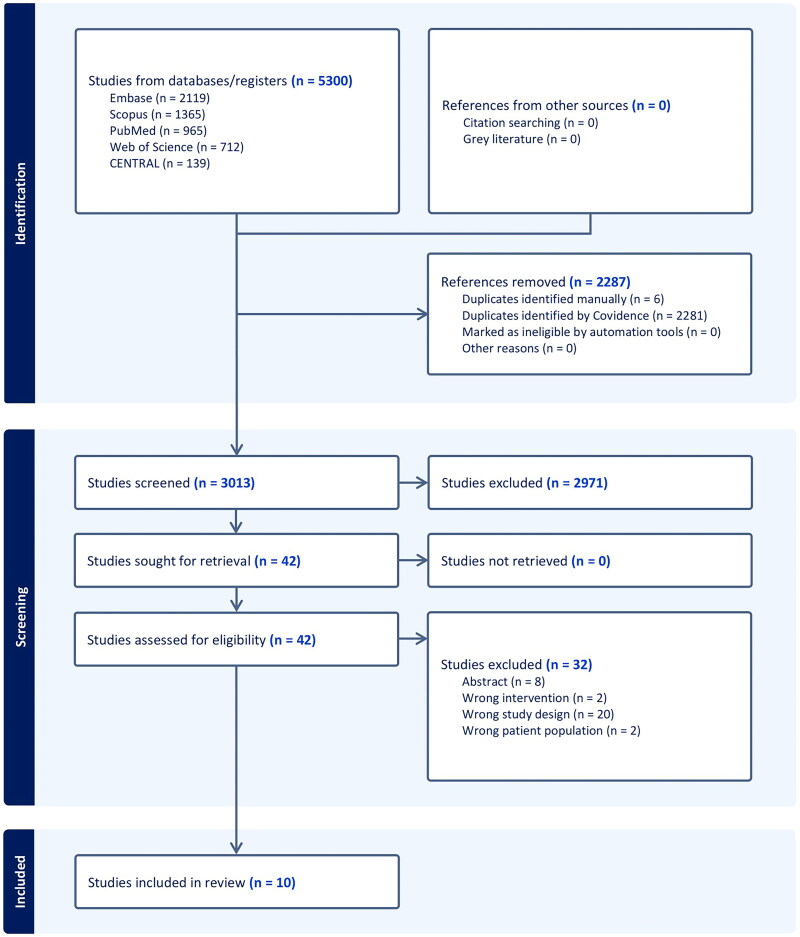
PRISMA flow chart of the screening process.

Five thousand and three hundred studies were screened and assessed based on their titles and abstracts during the search process. Forty-two articles moved on to full-text screening once (2287) duplicates and (2971) irrelevant studies were excluded. Finally, we included 10 RCTs (Figure 1).

### 3.2. Characteristics of included studies

Ten randomized clinical trials involving 18,355 adults with AKI were included in our analysis, comprising 10,191 (55.5%) men and 8164 (44.5%) women. The studies were conducted in China, the USA, Germany, and the UK. The follow-up period ranged from seven days to one year. The included participants’ comorbidities, baseline characteristics, and details of the included RCTs are detailed in Tables 1 and 2.

**Table 1.** Summary characteristics of the included RCTs.

Study ID	Study design	Country	Number of centers	Registry number	Total participants	Intervention			Control		Renal replacement therapy duration/dependency	Main inclusion criteria	Primary outcome	Follow-up duration
						Name	(Type: alert or email or message or tool or …)	Description	Name	Description				
Li 2024	Single-center, double-blind, parallel-group randomized clinical trial	China	1	NCT03736304	2208	Alert group	Message	The alarm system generated randomization automatically and texted doctors’ cell phones. The message contained an AKI alarm as well as a care package that comprised adjustments for dialysis and antimicrobial medicine dosage as needed, as well as optimization of hemodynamics and cessation of unnecessary nephrotoxic medication	Usual care	The alert system generated randomization automatically and did not send messages to usual care group	7 and 90 days, dialysis dependency at 90 days	Inpatient adults aged 18 years or older with AKI	Change of eGFR	7 days
Iwers 2023	Two-arm, prospective, cluster-randomized, controlled trial	Germany	1	DRKS-IDDRKS00017751	200	Intervention group	Alert	An internal email containing details regarding the patient’s identification, department, creatinine rise, and AKI stage was sent to the study’s investigator	Routine Care Group	Potential AKI was indicated by a lamp icon in the SAP work system of the hospital	90 days	Patients aged 18 years or more with AKI	Change of eGFR	12 months
Wilson 2023	Pragmatic, open-label, parallel group randomized controlled trial	USA	4	NCT02771977	5060	Alert group	Alert	A drug-specific alert, informing the provider of the presence of AKI as well as recent exposure to a potentially nephrotoxic agent, will be fired	Usual care	No alert will be fired	14 days of randomization	Adults aged 18 years or more with AKI	AKI progression, receipt of dialysis, or death	14 days
Wilson 2021	Double blinded, multicenter, parallel, randomized controlled trial	USA	0	NCT02753751	6030	Alert group	Alert	Provider’s will receive a ‘pop-up’ alert in the electronic health record until AKI is documented in the problem list or AKI resolves, the alert includes a link to an AKI order set with choices for kidney imaging, blood and urine testing	Usual care	No alert will be fired	14 days of randomization	Inpatient adults aged 18 or older with AKI	AKI progression, receipt of dialysis, or death	14 days
Haase-Fielitz 2020	Explorative randomized controlled trial	Germany	NA	DRKS00010530	52	Intensified treatment	Email	An early warning system for a rise in the serum creatinine concentration, immediate consultation of a specialist, and a set of measures include: Stop nephrotoxic medications or replace them with less nephrotoxic medications from the same substance class. Enhance hemodynamic performance; identify and address electrolyte and acid-base imbalances	Routine treatment	NA	NA	Patients aged 18 years or more who were treated on regular units for AKI	Renal recovery	NA
Selby 2019	Multicenter, pragmatic, stepped-wedge cluster randomized trial	UK	5	NA	NA	Intervention group	Alert	An electronic detection and alerting system for AKI, an AKI care bundle with individual components for assessment, investigation, and basic management of AKI (e.g., assess volume status and optimize blood pressure, treat sepsis, review medications and stop those contributing to AKI, perform urinalysis, refer AKI stage 3 patients with complications to nephrology or critical care outreach), and an educational program to increase health care workers’ awareness and knowledge of AKI	Control group	NA	NA	Patients older than 18 who were admitted to the hospital for at least one night during the study period and developed (AKI) during that stay	30-day mortality	NA
Thomas 2019	Pilot cluster randomized trial	UK	2	NCT02398682	1141	Intervention group	Alert	It sends a warning or ‘Alert’ about the test to our team of kidney experts, The Outreach team will quickly determine a reliable diagnosis of the underlying etiology of AKI, which will include: (enhanced evaluation of volume status, standardized urine dipstick usage, appropriate research on sepsis, Ultrasound done immediately with a possible blockage). And prompt ‘nephrotoxic’ drug cessation, treating the underlying cause of AKI promptly, and preventing AKI recurrence	Standard care	These patients will receive good standard care (active comparator), but none of the interventions listed for the Intervention group	30 days	Adult (more than 18 years) patient with an alert (Stages 1–3) due to AKI detected from a serum creatinine	Death within 30 days	365 days
Wu 2018	Prospective, randomized, controlled trial	China	1	NCT02793167	875	E-alert	Message	An AKI alert will be sent to the doctor in charge. Prior to alerting, the system screened adult patients who were at least 18 years old. The method would compare the serum creatinine values upon hospitalization, taking into account the outcomes of any prior hospital stays or outpatient visits. Then, using the Kidney Disease: Improving Global Outcomes criteria, the system diagnosed AKI	Non-e-alert	Patients will receive standard clinical care by the doctor in charge	Stage 3, dependent upon renal replacement therapy (discharge)	Adult patients aged ≥18 years with AKI	Adverse events during hospitalization	365 days
Wilson 2015	Investigator-masked, parallel-group, randomized controlled trial	USA	1	NCT01862419	2393	Alert group	Alert	Text page sent to patient’s covering provider and unit pharmacist informing them of the presence of AKI as detected by changes in serum creatinine, and the alert indicates who is responsible for performing the proper diagnostic and therapeutic actions	Usual care	No alert will be provided to the patient’s covering provider or unit pharmacist	7 days of randomization	Adults aged 18 years or older who were in hospital with stage 1 or greater AKI	Change in creatinine, dialysis, and death	30 days
McCoy 2012	Prospective, randomized, controlled trial	USA	1	NA	396	Intervention group	Alert	For all currently admitted, eligible patients, the surveillance view shows patient details like demographics, providing service, and hospital location. It also shows the most recent creatinine values and alerts about declining or improving renal function, enabling pharmacists to identify patients at high risk for harm	Control group	Clinical decision support alone	NA	Adult patients who experienced 0.5 mg/dl change in serum creatinine levels for more than 48 h after hospitalization	AKI-related ADEs	NA

NA: not available; AKI: acute kidney injury; eGFR: estimated glomerular filtration rate; SAP: systems applications and products.

**Table 2.** Baseline characteristics of the participants.

Study +ID	Number of patients in each group		Age (years), Mean (*SD*)		Gender (male), N. %		BMI, Mean (*SD*)		Smoking, N. %		Hospital admission at ICU, N. %		AKI grade, N. %						Comorbidities, N. %															
	Intervention	Control	Intervention	Control	Intervention	Control	Intervention	Control	Intervention	Control	Intervention	Control	1		2		3		Diabetes		Hypertension		Ischemic heart disease		Liver disease		Cerebrovascular disease		COPD		Congestive heart failure		Chronic kidney disease	
													Intervention	control	Intervention	control	Intervention	control	Intervention	control	Intervention	control	Intervention	control	Intervention	control	Intervention	control	Intervention	control	Intervention	control	Intervention	control
Li 2024	1123	1085	63.33 (13.53%)	64.33 (13.53%)	776 (69.1%)	784 (72.3%)	NA	NA	277 (24.7%)	269 (24.8%)	701 (62.4%)	637 (58.7%)	NA	NA	NA	NA	NA	NA	256 (22.8%)	219 (20.2%)	586 (52.2%)	568 (52.4%)	9 (34.6%)	8 (30.8%)	107 (9.5%)	83 (7.7%)	205 (18.3%)	217 (20.0%)	44 (3.9%)	38 (3.5%)	226 (20.1%)	212 (19.5%)	237 (21.1%)	250 (23.0%)
Iwers 2023	100	100	76 (13.53%)	78 (10.53%)	52 (52%)	56 (56%)	28.2 (6.46%)	27.9 (5.86%)	13 (13%)	5 (5%)	NA	NA	90 (90%)	89 (89%)	9 (9%)	9 (9%)	1 (1%)	2 (2%)	46 (46%)	41 (41%)	84 (84%)	76 (76%)	NA	NA	NA	NA	NA	NA	18 (18%)	12 (12%)	75 (75%)	72 (72%)	65 (67%)	67 (70.5%)
Wilson 2023	2532	2528	70 (16.31%)	70 (16.31%)	1291 (51%)	1315 (52%)	NA	NA	NA	NA	560 (22%)	598 (24%)	2279 (90%)	2248 (89%)	191 (7.5%)	230 (9.1%)	56 (2.2%)	47 (1.9%)	967 (38%)	928 (37%)	1710 (68%)	1726 (68%)	NA	NA	310 (12%)	359 (14%)	N/A	N/A	776 (31%)	762 (30%)	827 (33%)	784 (31%)	671 (27%)	616 (24%)
Wilson 2021	3059	2971	70.566 (16.83%)	70.766 (16.46%)	1618 (52.9%)	1530 (51.5%)	NA	NA	NA	NA	962 (31.4%)	961 (32.3%)	NA	NA	NA	NA	NA	NA	1287 (42.1%)	1197 (40.3%)	2510 (82.1%)	2434 (81.9%)	68 (34%)	64 (32.7%)	458 (15.0%)	397 (13.4%)	N/A	N/A	1049 (34.3%)	1015 (34.2%)	1351 (44.2%)	1307 (44.0%)	1163 (38.0%)	1127 (37.9%)
Haase-Fielitz 2020	26	26	69.33 (13.33%)	71.33 (14.9%)	16 (61.5%)	18 (69.2%)	27.8 (5.6%)	26.2 (4.6%)	NA	NA	NA	NA	19 (73.1%)	17 (65.4%)	5 (19.2%)	4 (15.4%)	2 (7.7%)	5 (19.2%)	11 (42.3%)	6 (23.1%)	22 (84.6%)	20 (76.9%)	NA	NA	10 (38.5%)	12 (46.2%)	NA	NA	3 (11.5%)	3 (11.5%)	NA	NA	NA	NA
Selby 2019	NA	NA	NA	NA	NA	NA	NA	NA	NA	NA	NA	NA	NA	NA	NA	NA	NA	NA	NA	NA	NA	NA	NA	NA	NA	NA	NA	NA	NA	NA	NA	NA	NA	NA
Thomas 2019	744	397	69.4 (16.6%)	72.6 (15.3%)	435 (58%)	182 (46%)	NA	NA	NA	NA	NA	NA	553 (74%)	290 (73%)	125 (17%)	68 (17%)	66 (9%)	39 (10%)	NA	NA	NA	NA	NA	NA	NA	NA	NA	NA	NA	NA	NA	NA	NA	NA
Wu 2018	467	408	62 (13.4%)	63.3 (13.4%)	298 (63.8%)	270 (66.2%)	NA	NA	NA	NA	NA	NA	NA	NA	NA	NA	NA	NA	NA	NA	NA	NA	NA	NA	NA	NA	NA	NA	NA	NA	NA	NA	NA	NA
Wilson 2015	1201	1192	60 (17%)	61 (16%)	670 (56%)	655 (55%)	NA	NA	NA	NA	365 (30%)	357 (30%)	NA	NA	NA	NA	NA	NA	352 (29%)	370 (31%)	NA	NA	NA	NA	164 (14%)	181 (15%)	142 (12%)	126 (11%)	NA	NA	386 (32%)	373 (31%)	323 (27%)	314 (26%)
McCoy 2012	200	196	60.7 (16.8%)	58.3 (15.7%)	106 (53%)	119 (60.7%)	NA	NA	NA	NA	NA	NA	NA	NA	NA	NA	NA	NA	83 (41.5%)	70 (35.7%)	134 (67%)	122 (62.2%)	NA	NA	8 (4%)	9 (4.6%)	29 (14.5%)	23 (11.7%)	NA	NA	52 (26%)	48 (24.5%)	NA	NA

NA: not available; *SD*: standard deviation; BMI: body mass index; ICU: intensive care unit; AKI: acute kidney injury; CDSS: clinical decision support systems: COPD: chronic obstructive pulmonary disease.

### 3.3. Risk of bias and certainty of evidence

**Figure 2.**
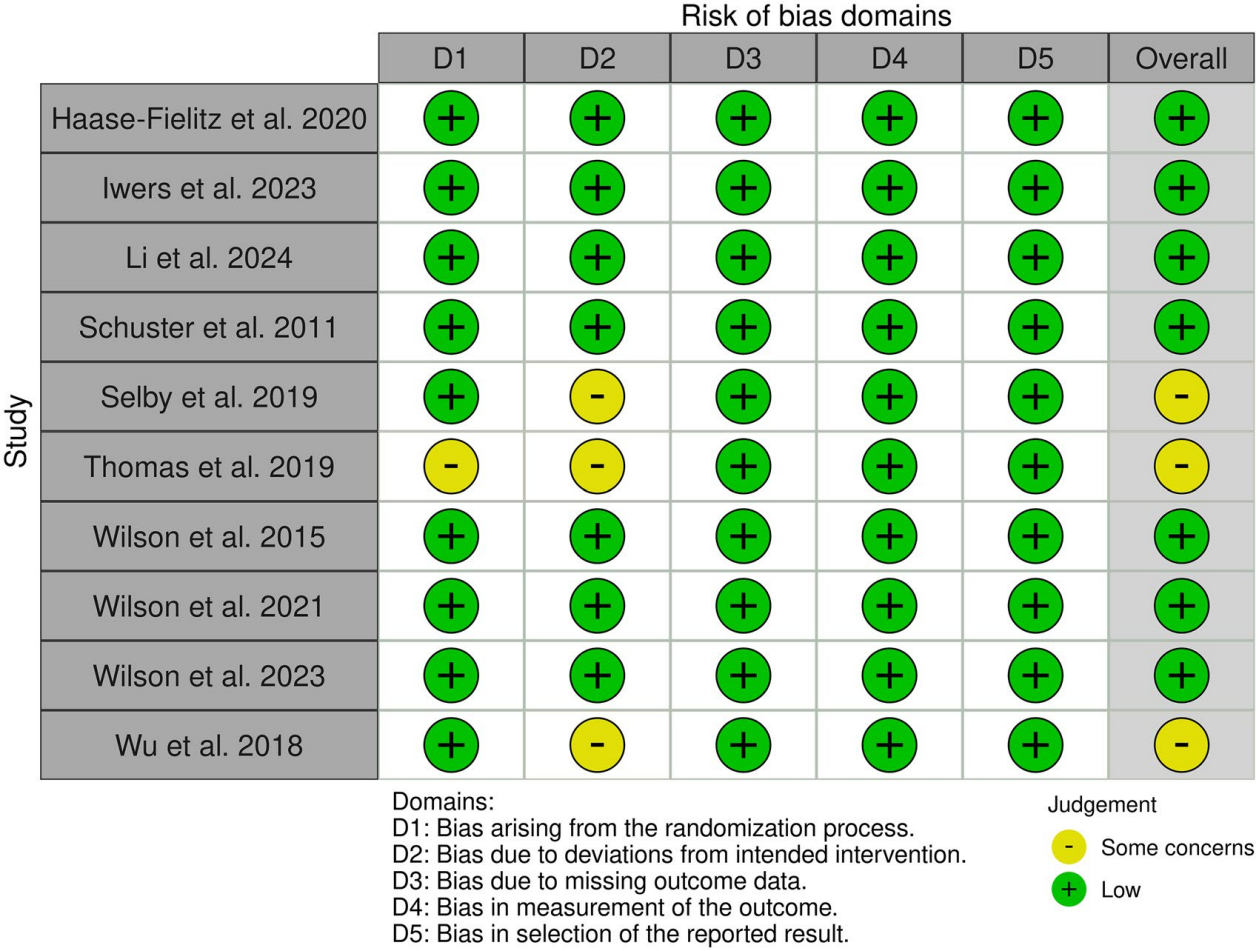
Quality assessment of risk of bias in the included trials. A schematic representation of risks (low = red, unclear = yellow, and high = red) for specific types of biases of each of the studies in the review.

Seven RCTs had a low risk of overall bias, and three RCTs showed some concerns mainly attributed to the unclear risk of reporting bias due to the randomization process and deviations from the intended intervention (Figure 2). Certainty of evidence is outlined in a GRADE evidence profile (Table 3).

**Table 3.** GRADE evidence profile.

Certainty assessment							Summary of findings				
Participants (studies) follow-up	Risk of bias	Inconsistency	Indirectness	Imprecision	Publication bias	Overall certainty of evidence	Study event rates (%)		Relative effect (95% CI)	Anticipated absolute effects	
							With [usual care]	With [CDSS]		Risk with [usual care]	Risk difference with [CDSS]
All-cause mortality											
17,959 (8 RCTs)	Not serious	Not serious	Not serious	Not serious	None	⊕⊕⊕⊕ High	1253/8707 (14.4%)	1322/9252 (14.3%)	RR 1.00 (0.93–1.07)	144 per 1000	0 fewer per 1000 (from 10 fewer to 10 more)
Renal replacement therapy											
18,355 (9 RCTs)	Not serious	Not serious	Not serious	Not serious	None	⊕⊕⊕⊕ High	530/8903 (6.0%)	609/9452 (6.4%)	RR 1.11 (0.99–1.24)	60 per 1000	7 more per 1000 (from 1 fewer to 14 more)
Hyperkalemia											
1589 (3 RCTs)	Serious^a^	Not serious	Not serious	Very serious^b^	None	⊕◯◯◯ Very low	13/619 (2.1%)	5/970 (0.5%)	RR 0.27 (0.10–0.73)	21 per 1000	15 fewer per 1000 (from 19 fewer to 6 fewer)
eGFR											
2460 (3 RCTs)	Not serious	Not serious	Not serious	Serious^c^	None	⊕⊕⊕◯ Moderate	1211	1249	–	–	MD 1.97 higher (0.47 higher to 3.48 higher)
Re-hospitalization											
5307 (3 RCTs)	Not serious	Not serious	Not serious	Not serious	None	⊕⊕⊕⊕ High	385/2653 (14.5%)	348/2654 (13.1%)	RR 0.90 (0.79–1.03)	145 per 1000	15 fewer per 1000 (from 30 fewer to 4 more)
Creatinine											
4801 (3 RCTs)	Not serious	Not serious	Not serious	Not serious	None	⊕⊕⊕⊕ High	2377	2424	–	–	MD 0 (0.04 lower to 0.04 higher)
AKI in discharge											
3335 (4 RCTs)	Serious^d^	Very serious^e^	Not serious	Serious^c^	None	⊕◯◯◯ Very low	464/1619 (28.7%)	770/1716 (44.9%)	RR 1.39 (0.94–2.05)	287 per 1000	112 more per 1000 (from 17 fewer to 301 more)
Renal recovery											
3135 (3 RCTs)	Serious^f^	Not serious	Not serious	Not serious	None	⊕⊕⊕◯ Moderate	790/1519 (52.0%)	831/1616 (51.4%)	RR 0.99 (0.93–1.06)	520 per 1000	5 fewer per 1000 (from 36 fewer to 31 more)
Progression of AKI											
14,439 (4 RCTs)	Not serious	Not serious	Not serious	Not serious	None	⊕⊕⊕⊕ High	1280/6981 (18.3%)	1310/7458 (17.6%)	RR 0.98 (0.92–1.05)	183 per 1000	4 fewer per 1000 (from 15 fewer to 9 more)
Length of hospital stay											
39,950 (6 RCTs)	Not serious	Very serious^e^	Not serious	Not serious	None	⊕⊕◯◯ Low	21,918	18,032	–	–	MD 0.22 lower (1.31 lower to 0.86 higher)
Hospital cost											
13,298 (3 RCTs)	Not serious	Not serious	Not serious	Extremely serious^c^	None	⊕◯◯◯ Very low	6584	6714	–	–	MD 826.46 higher (110.14 lower to 1763.06 higher)
Patients received NASIDs											
15,943 (6 RCTs)	Not serious	Serious^g^	Not serious	Serious^c^	None	⊕⊕◯◯ Low	1212/7902 (15.3%)	1064/8041 (13.2%)	RR 0.81 (0.65–1.00)	153 per 1000	29 fewer per 1000 (from 54 fewer to 0 fewer)
Patients received aminoglycosides											
10,631 (3 RCTs)	Not serious	Not serious	Not serious	Serious^c^	None	⊕⊕⊕◯ Moderate	126/5248 (2.4%)	99/5383 (1.8%)	RR 0.78 (0.60–1.00)	24 per 1000	5 fewer per 1000 (from 10 fewer to 0 fewer)
Patients received ACEi and ARBs											
15,891 (5 RCTs)	Not serious	Not serious	Not serious	Not serious	None	⊕⊕⊕⊕ High	2148/7876 (27.3%)	2187/8015 (27.3%)	RR 1.01 (0.96–1.06)	273 per 1000	3 more per 1000 (from 11 fewer to 16 more)
Patients received contrast											
10,831 (4 RCTs)	Not serious	Not serious	Not serious	Not serious	None	⊕⊕⊕⊕ High	461/5348 (8.6%)	474/5483 (8.6%)	RR 1.01 (0.91–1.13)	86 per 1000	1 more per 1000 (from 8 fewer to 11 more)
Patients received fluid											
10,831 (4 RCTs)	Not serious	Very serious^e^	Not serious	Serious^c^	None	⊕◯◯◯ Very low	1524/5348 (28.5%)	1839/5483 (33.5%)	RR 1.13 (0.95–1.36)	285 per 1000	37 more per 1000 (from 14 fewer to 103 more)
Patients underwent renal ultrasound											
4653 (3 RCTs)	Not serious	Very serious^e^	Not serious	Very serious^c^	None	⊕◯◯◯ Very low	103/2303 (4.5%)	165/2350 (7.0%)	RR 1.99 (0.69–5.78)	45 per 1000	44 more per 1000 (from 14 fewer to 214 more)
Nephrologist consultation											
16,818 (7 RCTs)	Not serious	Serious^g^	Not serious	Serious^c^	None	⊕⊕◯◯ Low	1440/8310 (17.3%)	1555/8508 (18.3%)	RR 1.13 (0.96–1.33)	173 per 1000	23 more per 1000 (from 7 fewer to 57 more)

CI: confidence interval; MD: mean difference; RR: risk ratio.

^a^Thomas et al. had an overall concern of bias with 65% of pooled analysis weight.

^b^A wide confidence interval that does not exclude the risk of appreciable harm/benefit with a low number of events.

^c^A wide confidence interval that does not exclude the risk of appreciable harm/benefit.

^d^Wu et al. had an overall concern of bias with 31.7% of pooled analysis weight.

^e^*I*^2^ > 75%.

^f^Wu et al. had an overall concern of bias with 27% of pooled analysis weight.

^g^*I*^2^ > 50%.

### 3.4. Primary outcomes: all-cause mortality and renal replacement therapy

**Figure 3.**
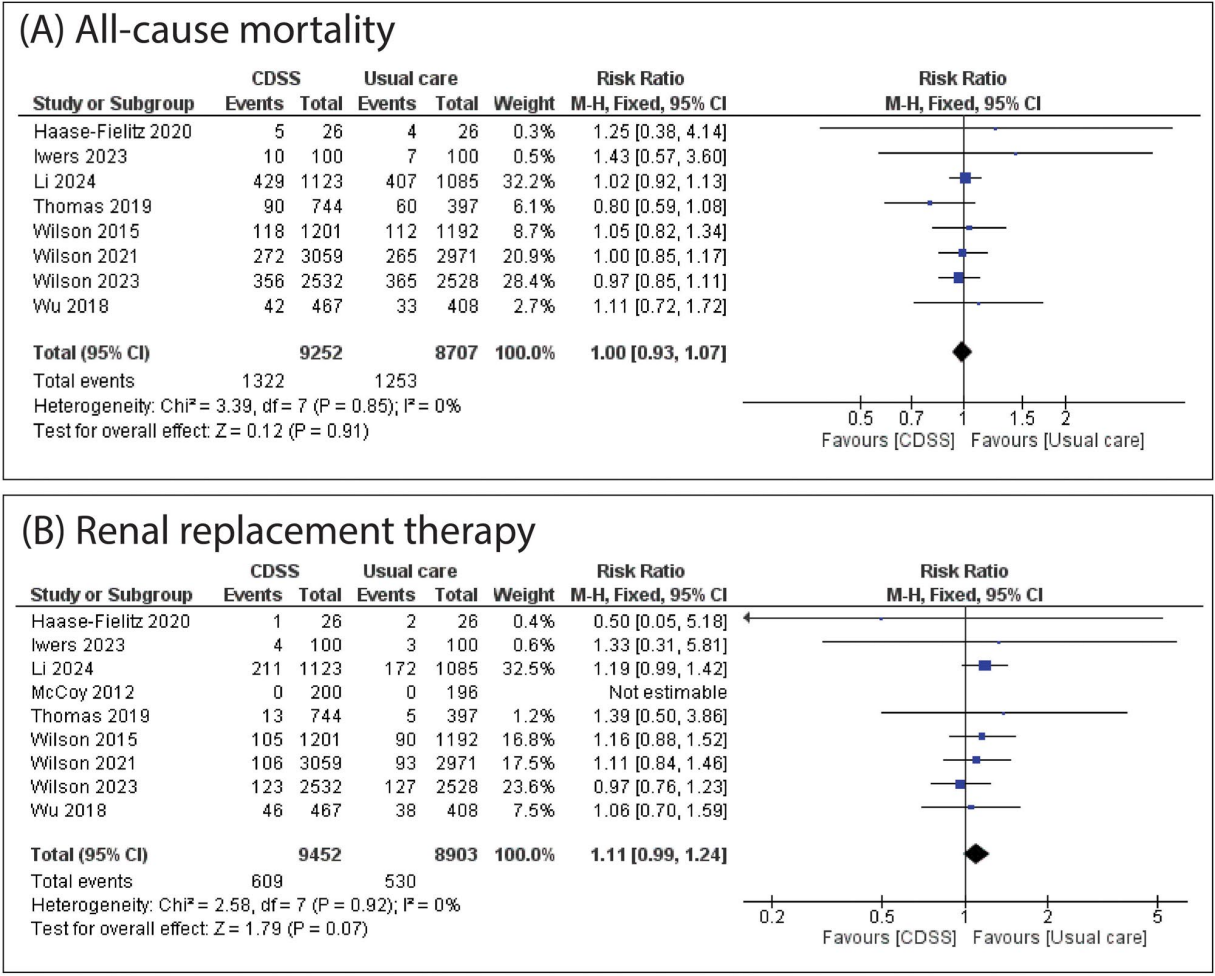
Forest Plot of the primary outcomes (all-cause mortality and renal replacement therapy), RR: risk ratio, CI: confidence interval.

There was no significant difference between CDSS and usual care in all-cause mortality (RR: 1.00 with 95% CI [0.93, 1.07], *p* = 0.91) and renal replacement therapy (RR: 1.11 with 95% CI [0.99, 1.24], *p* = 0.07). The pooled studies were homogenous in all-cause mortality and renal replacement therapy (*I*^2^ = 0%, *p* = 0.85) and (*I*^2^ = 0%, *p* = 0.92), respectively (Figure 3).

### 3.5. Secondary outcomes

#### 3.5.1. Clinical outcomes

**Figure 4.**
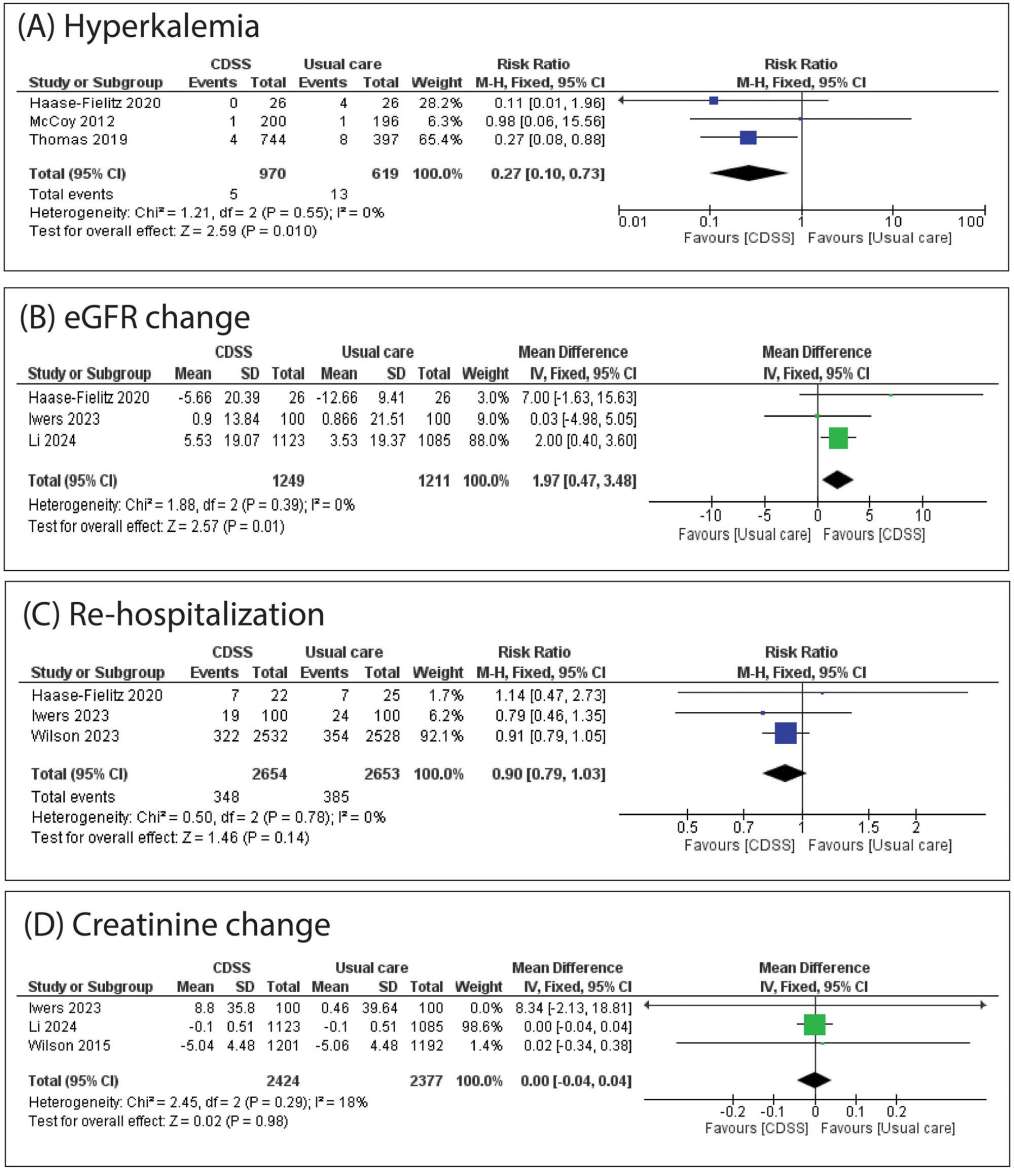
(A) Forest plot of the hyperkalemia; (B) forest plot of the eGFR; (C) forest plot of the re-hospitalization; (D) forest plot of the creatinine change. RR: risk ratio; CI: confidence interval.

CDSS was significantly associated with a decreased incidence of hyperkalemia (RR: 0.27 with 95% CI [0.10, 0.73], *p* = 0.01) (Figure 4(A)) and increased eGFR change (MD: 1.97 with 95% CI [0.47, 3.48], *p* = 0.01) (Figure 4(B)). However, there was no significant difference between CDSS and usual care in re-hospitalization (RR: 0.90 with 95% CI [0.79, 1.03], *p* = 0.14) (Figure 4(C)), creatinine change (MD: 0.00 with 95% CI [−0.04, 0.04], *p* = 0.98) (Figure 4(D)), AKI on discharge (RR: 1.39 with 95% CI [0.94, 2.05], *p* = 0.10), renal recovery (RR: 0.99 with 95% CI [0.93, 1.06], *p* = 0.76), progression of AKI (RR: 0.98 with 95% CI [0.92, 1.05], *p* = 0.65), length of hospital stay (MD: −0.22 with 95% CI [−1.31, 0.86], *p* = 0.69), and hospital cost (MD: 826.46 with 95% CI [−110.14, 1763.06], *p* = 0.08) (Figures S1–S5).

**Figure 5.**
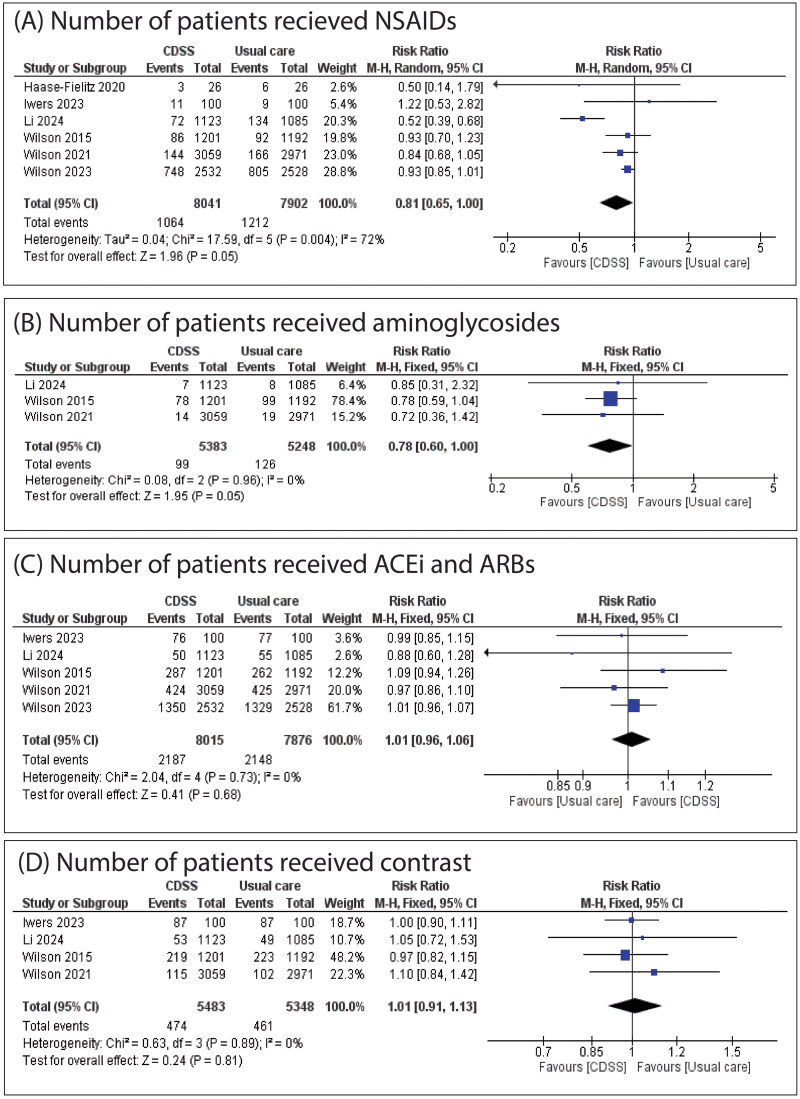
(A) Forest plot of the number of patients who received NSAIDs; (B) forest plot of the number of patients who received aminoglycosides; (C) forest plot of number of patients who received ACEi and ARBs; (D) forest plot of number of patients received contrast. RR: risk ratio; CI: confidence interval.

The pooled studies were homogenous in hyperkalemia (*I*^2^ = 0%, *p* = 0.55), eGFR change (*I*^2^ = 0%, *p* = 0.39), re-hospitalization (*I*^2^ = 0%, *p* = 0.78), creatinine change (*I*^2^ = 18%, *p* = 0.29), renal recovery (*I*^2^ = 5%, *p* = 0.35), progression of AKI (*I*^2^ = 34%, *p* = 0.21), and hospital cost (*I*^2^ = 12%, *p* = 0.32). However, pooled studies were heterogeneous in AKI in discharge (*I*^2^ = 89%, *p* < 0.00001) and length of hospital stay (*I*^2^ = 97%, *p* < 0.00001). Regarding AKI in discharge, heterogeneity was best resolved by excluding Li 2024 and Wu 2018 (*I*^2^ = 41%, *p* = 0.18) and (*I*^2^ = 0%, *p* = 0.42). Regarding length of hospital stay, heterogeneity was not resolved by sensitivity analysis, as shown in Table S2.

#### 3.5.2. Management outcomes

There was no significant difference between CDSS and usual care regarding the number of patients who received NSAIDs (RR: 0.81 with 95% CI [0.65, 1.00], *p* = 0.05) (Figure 5(A)), the number of patients who received aminoglycosides (RR: 0.78 with 95% CI [0.60, 1.00], *p* = 0.05) (Figure 5(B)), the number of patients who received ACEi or ARBs (RR: 1.01 with 95% CI [0.96, 1.06], *p* = 0.68) (Figure 5(C)), contrast (RR: 1.01 with 95% CI [0.91, 1.13], *p* = 0.81) (Figure 5(D)), fluids (RR: 1.13 with 95% CI [0.95, 1.36], *p* = 0.17), number of patients who underwent renal ultrasonography (RR: 1.99 with 95% CI [0.69, 5.78], *p* = 0.20), and nephrologist consultation (RR: 1.13 with 95% CI [0.96, 1.33], *p* = 0.14) (Figures S6–S8).

The pooled studies were homogenous in the number of patients who received aminoglycosides (*I*^2^ = 0%, *p* = 0.96), ACEi and ARBs (*I*^2^ = 0%, *p* = 0.73), contrast (*I*^2^ = 0%, *p* = 0.89). However, pooled studies were heterogeneous in the number of patients on NSAIDs (*I*^2^ = 72%, *p* = 0.004), number of patients who received fluids (*I*^2^ = 88%, *p* < 0.0001), number of patients who underwent renal ultrasonography (*I*^2^ = 93%, *p* < 0.00001), and nephrologist consultation (*I*^2^ = 70%, *p* = 0.003). Regarding the number of patients on NSAIDs, heterogeneity was best resolved by excluding Li 2024 (*I*^2^ = 0%, *p* = 0.74). Regarding the number of patients who underwent renal ultrasonography, heterogeneity was best resolved by excluding Li 2024 (*I*^2^ = 0%, *p* = 0.53). Regarding the number of patients who received fluids, heterogeneity was best resolved by excluding Li 2024 (*I*^2^ = 5%, *p* = 0.35). Regarding nephrologist consultation, sensitivity analysis did not resolve heterogeneity, as shown in Table S2.

## 4. Discussion

We observed comparable outcomes between CDSS and usual care regarding all-cause mortality and renal replacement therapy. CDSS was significantly associated with a decreased incidence of hyperkalemia and an increased change in eGFR. However, there was no significant difference between CDSS and usual care regarding re-hospitalization, creatinine change, AKI at discharge, renal recovery, progression of AKI, length of hospital stay, and hospital cost.

The previous Zhao et al. [16] meta-analysis encompassed seven studies and involved 32,846 participants. The results concerning renal replacement therapy, length of hospital stay, and progression of AKI were similar. However, the results relating to mortality differed. The study highlighted that implementing CDSS reduced overall mortality (OR 0.86; 95% CI 0.75–0.99; *p* = 0.040, *n* = 5 studies), with significant heterogeneity. Notably, that outcome included only one RCT, Selby et al. [17]. It is also essential to highlight that our meta-analysis did not include Selby et al. in some outcomes. However, it was included in Zhao et al.’s meta-analysis because the authors did not report crude data.

Interestingly, this RCT had the most significant number of participants (24,059) and reported comparable 30-day mortality results for the pooled analysis. However, the multilevel logistic regression results for mortality showed a 13% increase in mortality in winter compared to spring. There was also an increase in mortality with older age, a decrease in mortality by 14% in females, and a higher mortality associated with a higher Charlson comorbidity score [17].

Moreover, it is also worth mentioning that, according to Chávez-Íñiguez et al. [18], patients with AKI and persistent hyperkalemia were correlated with the need for kidney replacement therapy, while those with changes from normal potassium to hyperkalemia were associated with death. In our study, as highlighted in the following paragraphs, there was a reduction in the incidence of hyperkalemia. These results emphasize that mortality should not be viewed as the sole determinant of primary outcomes, instead considering all aspects of the patient’s health.

Also, in our study, renal replacement therapy did not differ between groups, irrespective of whether a CDSS was in place. This could potentially be attributed to the fact that patients who require renal replacement therapy present with the most severe form of AKI. Consequently, integrating renal replacement therapy into the ongoing management of critically ill patients could lead to an escalation in complexity and cost [19]. Therefore, deciding to initiate a critically ill patient on renal replacement therapy must balance the increased bedside workload and resource utilization and the preferences for care expressed by the patient and their family [20].

A significant finding of this study was that the CDSS was notably associated with a decrease in the incidence of hyperkalemia and an increase in eGFR change. Simultaneously, CDSS was insignificantly linked with a decline in the number of patients who received NSAIDs and aminoglycosides. However, due to the limited number of studies, particularly for NSAIDs and aminoglycosides, it is challenging to determine whether the reduction in hyperkalemia and increase in eGFR were results only of not taking these medications.

According to the guidelines, NSAIDs should be avoided in people with eGFR < 30 mL/min per 1.73 m^2^, and prolonged use should be avoided in those with eGFR between 30 and 59 mL/min per 1.73 m^2^ [21]. Furthermore, NSAIDs are known to cause hyperkalemia [22], not to mention the nephrotoxic effect of aminoglycosides, which require dose adjustment in cases of renal failure [23]. These results, however, indicate the usefulness of CDSS in the early detection of patients with higher risk of AKI and in tailoring their medication strategy.

However, a comprehensive approach to the work process is essential. This perspective has been explored by Marcilly et al. [24] in their DetecIP project, which yielded several noteworthy observations. Preliminary findings from geriatric and cardiology units indicate that despite the overall similarities in work processes, the management of iatrogenic risks of hyperkalemia/AKI is influenced by the availability and location of physicians and clinical pharmacists. Pharmacists conduct pharmaceutical analyses of orders in a relatively similar manner across units, irrespective of the use of the pharmacist CDSS. However, the physical proximity between residents and pharmacists varies between units. In the cardiology clinic, the shared office space facilitates cooperation, whereas in the geriatric unit, the pharmacist’s location outside the ward hinders collaboration. Consequently, the authors suggest that implementing pharmacist CDSS may yield different outcomes in cardiology and geriatrics units for AKI/hyperkaliemia prevention or management due to these variations [24]. Therefore, future trials investigating CDSS may also explain the work process in which the CDSS was implemented for a holistic approach analysis.

## 5. Strength and limitations

Our study demonstrates several strengths. First, the comprehensive literature search covered five databases until 21st January 2024, to ensure that relevant studies are included, minimizing selection bias. Second, the study adheres to a robust research design by including only RCTs. Third, including ten RCTs with over 18,355 adults enhances the statistical power of the analysis.

Still, this meta-analysis was subject to a few limitations. First, we primarily reported the pooled analysis, and it was not possible to conduct a subgroup analysis based on the CDSS form mentioned above or based on follow-up. This was mainly due to the various implemented CDSS forms and inconsistent follow-up periods. Still, the pooled analysis for primary outcomes showed homogeneous results. Second, some outcomes showed heterogeneity, such as AKI at discharge, length of hospital stay, number of patients who received NSAIDs, nephrologist consultation, and number of patients who received fluids. Therefore, we performed a sensitivity analysis and resolved all heterogeneous results except for nephrologist consultation and length of hospital stay. Third, our analysis relies on pooled data from diverse studies, each with inherent limitations. These limitations are shared across the included studies. Fourth, we could not appropriately assess asymmetry in funnel plots due to the small number of studies (<10) included in our analysis.

## 6. Implications for future research

The decision to implement a CDSS in a healthcare facility should be based on a process gap that needs to be filled or supported. In this study, we have provided several forms of CDSS, including systems for recognizing high-risk patients with AKI for clinical pharmacist intervention [25], early warning systems for a rise in serum creatinine concentration, immediate consultation with a specialist, and issuing a patient kidney passport [26]. We also included a care bundle involving fluid administration, potentially nephrotoxic medication discontinuation, and nephrology consultation [27]. Each form of CDSS was designed for specific purposes, and its usefulness should be limited within the scope of these purposes. For instance, CDSS were generally observed to improve AKI care delivery. However, improving AKI care delivery does not necessarily translate exclusively to lower mortality [17]. Still, as observed in our analysis, it can also lead to improved clinical functions, such as eGFR and reduced unwanted side effects, such as hyperkalemia.

Finally, while the hospital cost seems insignificantly higher with CDSS, the length of hospital stay was insignificantly shorter. However, considering the large number of patients with AKI, i.e., ∼750,000 patients per year in the United States [28], this could account for a substantial health-economic benefit. We recommend that healthcare facilities conduct a cost-effectiveness analysis before implementing the CDSS system, considering direct operational and maintenance costs and indirect costs associated with administrative functions for healthcare professionals.

## 7. Conclusion

CDSS did not prevent all-cause mortality or decrease the need for renal replacement therapy in patients with AKI but reduced the incidence of hyperkalemia and increased eGFR. Tailoring CDSS to the specific needs of healthcare facilities can further amplify its impact. However, additional research is essential to ensure long-term sustainability and successful real-world implementation, especially when standardizing CDSS across multiple facilities.

## Supplementary Material

Supplementary material.docx

## Data Availability

The data is available upon reasonable request from the corresponding author.
