# Supplementary material for: The effect of clinical decision support systems on clinical outcomes in acute kidney injury: a systematic review and meta-analysis of randomized controlled trials
Source: Ren Fail. 2024 Sep 9;46(2):2400552. doi: 10.1080/0886022X.2024.2400552 (PMC11389631; doi:10.1080/0886022X.2024.2400552)

**Supplementary material.**

**Title**.

The Effect of Clinical Decision Support Systems on Acute Kidney Injury Outcomes: A Systematic Review and Meta-Analysis of Randomized Controlled Trials

**Authors.**

Obieda Altobaishat^1^, Mohamed Abouzid^2^, Ahmed Mazen Amin^3^, Abdallah Bani-Salameh^1^, Mohammad Tanashat^4^, Omar Abdullah Bataineh^1^, Mustafa Turkmani^5,6^, Mohamed Abuelazm^7^, Muner M B Mohamed^8,9^.

**Affiliations.**

1. Faculty of Medicine, Jordan University of Science and Technology, Irbid, Jordan
2. Department of Physical Pharmacy and Pharmacokinetics, Faculty of Pharmacy, Poznan University of Medical Sciences, Rokietnicka 3 St., 60-806 Poznan, Poland
3. Faculty of Medicine, Mansoura University, Mansoura, Egypt
4. Faculty of Medicine, Yarmouk University, Irbid, Jordan
5. Faculty of Medicine, Michigan State University, East Lansing, Michigan, USA
6. Department of Internal Medicine, McLaren Health Care, Oakland, Michigan, USA
7. Faculty of Medicine Tanta University, Tanta, Egypt
8. Department of Nephrology, Ochsner Health System, New Orleans, LA, USA
9. Ochsner Clinical School, The University of Queensland, Brisbane, QLD, Australia

**Contents.**

**Tables.**

**Table S1: Search strategy.**

Date/ 21/01/2024

| Database | Search Terms | Search Field | Search Results |
| --- | --- | --- | --- |
| PubMed | ("Acute kidney injury" OR "AKI" OR "Acute kidney disease" OR "AKD" OR "Acute renal failure") AND ("Electronic alert" OR "E-alert" OR "E alert" OR "alarm" OR "alert" OR "reporting system" OR "warn" OR "Alarm monitor" OR "Clinical Alarms" OR "Physiologic Monitoring" OR "Point-of-Care Systems" OR "Reminder Systems" OR "Medication alert system" OR "Care bundle" OR "patient care bundle" OR "Decision support system" OR "clinical decision support system" OR "Computer-Assisted Drug Therapy" OR "Computerized decision support" OR "Computerized order entry system" OR "order entry system" OR "Electronic order entry system" OR "Electronic Health Record") | All Field | 965 |
| Cochrane | ("Acute kidney injury" OR "AKI" OR "Acute kidney disease" OR "AKD" OR "Acute renal failure") AND ("Electronic alert" OR "E-alert" OR "E alert" OR "alarm" OR "alert" OR "reporting system" OR "warn" OR "Alarm monitor" OR "Clinical Alarms" OR "Physiologic Monitoring" OR "Point-of-Care Systems" OR "Reminder Systems" OR "Medication alert system" OR "Care bundle" OR "patient care bundle" OR "Decision support system" OR "clinical decision support system" OR "Computer-Assisted Drug Therapy" OR "Computerized decision support" OR "Computerized order entry system" OR "order entry system" OR "Electronic order entry system" OR "Electronic Health Record") | All Field | 139 |
| WOS | ("Acute kidney injury" OR "AKI" OR "Acute kidney disease" OR "AKD" OR "Acute renal failure") AND ("Electronic alert" OR "E-alert" OR "E alert" OR "alarm" OR "alert" OR "reporting system" OR "warn" OR "Alarm monitor" OR "Clinical Alarms" OR "Physiologic Monitoring" OR "Point-of-Care Systems" OR "Reminder Systems" OR "Medication alert system" OR "Care bundle" OR "patient care bundle" OR "Decision support system" OR "clinical decision support system" OR "Computer-Assisted Drug Therapy" OR "Computerized decision support" OR "Computerized order entry system" OR "order entry system" OR "Electronic order entry system" OR "Electronic Health Record") | All Field | 712 |
| SCOPUS | ("Acute kidney injury" OR "AKI" OR "Acute kidney disease" OR "AKD" OR "Acute renal failure") AND ("Electronic alert" OR "E-alert" OR "E alert" OR "alarm" OR "alert" OR "reporting system" OR "warn" OR "Alarm monitor" OR "Clinical Alarms" OR "Physiologic Monitoring" OR "Point-of-Care Systems" OR "Reminder Systems" OR "Medication alert system" OR "Care bundle" OR "patient care bundle" OR "Decision support system" OR "clinical decision support system" OR "Computer-Assisted Drug Therapy" OR "Computerized decision support" OR "Computerized order entry system" OR "order entry system" OR "Electronic order entry system" OR "Electronic Health Record") | Title, Abstract, Keywords | 1365 |
| EMBASE | ("Acute kidney injury" OR "AKI" OR "Acute kidney disease" OR "AKD" OR "Acute renal failure") AND ("Electronic alert" OR "E-alert" OR "E alert" OR "alarm" OR "alert" OR "reporting system" OR "warn" OR "Alarm monitor" OR "Clinical Alarms" OR "Physiologic Monitoring" OR "Point-of-Care Systems" OR "Reminder Systems" OR "Medication alert system" OR "Care bundle" OR "patient care bundle" OR "Decision support system" OR "clinical decision support system" OR "Computer-Assisted Drug Therapy" OR "Computerized decision support" OR "Computerized order entry system" OR "order entry system" OR "Electronic order entry system" OR "Electronic Health Record") | All Field | 2119 |

**Table S2: Sensitivity analysis.**

| Outcome | No. of  Participants (/) | No. of  trials | Quantitative data synthesis | | | | Heterogeneity analysis | | |
| --- | --- | --- | --- | --- | --- | --- | --- | --- | --- |
|  |  |  | RR | 95% CI | Z value | p-value | df | p-value | I2 (%) |
| **AKI in discharge.** | | | | | | | | | |
| \| Haase-Fielitz 2020 \| \| --- \| | 1690/1593 | 3 | 1.45 | [0.95, 2.19] | 1.73 | 0.08 | 2 | 0.0001 | 92% |
| \| Iwers 2023 \| \| --- \| | 1616/1519 | 3 | 1.32 | [0.81, 2.16] | 1.11 | 0.27 | 2 | 0.0001 | 92% |
| \| **Li 2024** \| \| --- \| | 593/534 | 3 | 1.19 | [0.88, 1.62] | 1.12 | 0.26 | 2 | 0.18 | 41% |
| \| **Wu 2018** \| \| --- \| | 1249/1211 | 3 | 1.80 | [1.61, 2.00] | 10.57 | 0.00001 | 2 | 0.42 | 0% |
| **Length of hospital stay.** | | | | | | | | | |
| \| Iwers 2023 \| \| --- \| | 17932/21818 | 5 | -0.41 | [-1.55, 0.73] | 0.70 | 0.48 | 4 | 0.00001 | 98% |
| \| Li 2024 \| \| --- \| | 16909/20833 | 5 | -0.39 | [-1.57, 0.79] | 0.65 | 0.52 | 4 | 0.00001 | 98% |
| \| \| Selby 2019 \| \| --- \| \| \| --- \| --- \| | 8015/7876 | 5 | 0.03 | [-0.40, 0.46] | 0.14 | 0.89 | 4 | 0.03 | 62% |
| \| Wilson 2015 \| \| --- \| | 16831/20726 | 5 | -0.09 | [-1.34, 1.16] | 0.14 | 0.89 | 4 | 0.00001 | 98% |
| \| Wilson 2021 \| \| --- \| | 14973/18947 | 5 | -0.24 | [-1.58, 1.10] | 0.35 | 0.73 | 4 | 0.00001 | 97% |
| \| Wilson 2023 \| \| --- \| | 15500/19390 | 5 | -0.33 | [-1.55, 0.90] | 0.53 | 0.60 | 4 | 0.00001 | 97% |
| **Number of patients received NSAIDs.** | | | | | | | | | |
| \| Haase-Fielitz 2020 \| \| --- \| | 8015/7876 | 5 | 0.82 | [0.65, 1.02] | 1.80 | 0.07 | 4 | 0.002 | 76% |
| Iwers 2023 | 7941/7802 | 5 | 2.08 | [0.63, 0.99] | 2.08 | 0.04 | 4 | 0.002 | 76% |
| \| \| \| **Li 2024** \| \| --- \| \| \| --- \| --- \| \| \| --- \| --- \| --- \| | 6918/6817 | 5 | 0.92 | [0.85, 0.99] | 2.27 | 0.02 | 4 | 0.74 | 0% |
| \| Wilson 2015 \| \| --- \| | 6840/6710 | 5 | 0.78 | [0.59, 1.02] | 1.82 | 0.07 | 4 | 0.002 | 77% |
| \| Wilson 2021 \| \| --- \| | 4982/4931 | 5 | 0.79 | [0.59, 1.07] | 1.51 | 0.13 | 4 | 0.002 | 77% |
| \| Wilson 2023 \| \| --- \| | 5509/5374 | 5 | 0.76 | [0.57, 1.03] | 1.79 | 0.07 | 4 | 0.02 | 66% |
| **Nephrologist consultation.** | | | | | | | | | |
| \| Haase-Fielitz 2020 \| \| --- \| | 8482/8284 | 6 | 1.09 | [0.96, 1.23] | 1.28 | 0.20 | 5 | 0.04 | 58% |
| Iwers 2023 | 8408/8210 | 6 | 1.13 | [0.96, 1.34] | 1.49 | 0.14 | 5 | 0.002 | 74% |
| \| \| \| Li 2024 \| \| --- \| \| \| --- \| --- \| \| \| --- \| --- \| --- \| | 7385/7225 | 6 | 1.15 | [0.93, 1.41] | 1.28 | 0.20 | 5 | 0.002 | 73% |
| \| Wilson 2015 \| \| --- \| | 7307/7118 | 6 | 1.15 | [0.94, 1.40] | 1.38 | 0.17 | 5 | 0.001 | 75% |
| \| Wilson 2021 \| \| --- \| | 5449/5339 | 6 | 1.23 | [0.96, 1.56] | 1.67 | 0.09 | 5 | 0.003 | 72% |
| \| Wilson 2023 \| \| --- \| | 5976/5782 | 6 | 1.21 | [0.96, 1.53] | 1.64 | 0.10 | 5 | 0.001 | 75% |
| Wu 2018 | 8041/7902 | 6 | 1.06 | [0.93, 1.20] | 0.90 | 0.37 | 5 | 0.04 | 56% |
| **Number of patients underwent renal ultrasound.** | | | | | | | | | |
| \| Wilson 2015 \| \| --- \| | 1149/1111 | 2 | 2.93 | [0.18, 47.38 | 0.76 | 0.45 | 1 | 0.00001 | 97% |
| \| \| \| **Li 2024** \| \| --- \| \| \| --- \| --- \| \| \| --- \| --- \| --- \| | 1227/1218 | 2 | 1.06 | [0.83, 1.36] | 0.49 | 0.62 | 1 | 0.53 | 0% |
| \| Haase-Fielitz 2020 \| \| --- \| | 2324/2277 | 2 | 3.14 | [0.35, 28.03] | 1.03 | 0.31 | 1 | 0.00001 | 96% |
| **Number of patients received fluids.** | | | | | | | | | |
| Iwers 2023 | 1818/8210 | 3 | 1.14 | [0.94, 1.39] | 1.36 | 0.17 | 2 | 0.00001 | 92% |
| \| \| \| **Li 2024** \| \| --- \| \| \| --- \| --- \| \| \| --- \| --- \| --- \| | 4360/4263 | 3 | 1.05 | [0.97, 1.15] | 1.17 | 0.24 | 2 | 0.35 | 5% |
| \| Wilson 2015 \| \| --- \| | 4282/4156 | 3 | 1.22 | [1.06, 1.39] | 2.86 | 0.004 | 2 | 0.08 | 61% |
| \| Wilson 2021 \| \| --- \| | 2424/2377 | 3 | 1.13 | [0.88, 1.45] | 0.94 | 0.34 | 2 | 0.00001 | 91% |

RR: risk ratio; CI: confidence interval; df: degrees of freedom.

**Figures.**

Figure S1: Forest plot of AKI in discharge, CI: confidence interval.


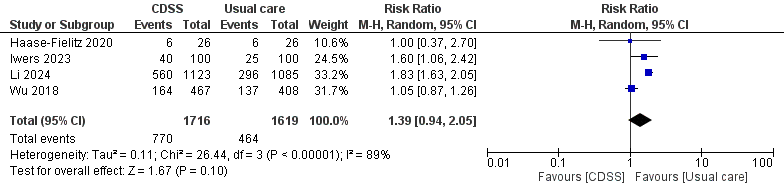


Figure S2: Forest plot of renal recovery, CI: confidence interval.


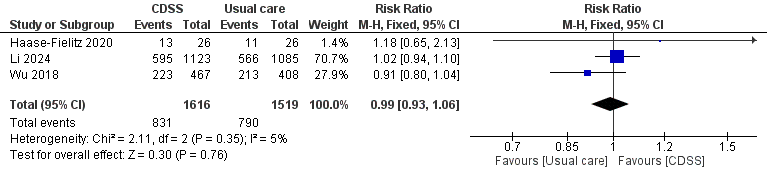


Figure S3: Forest plot of progression of AKI, CI: confidence interval.


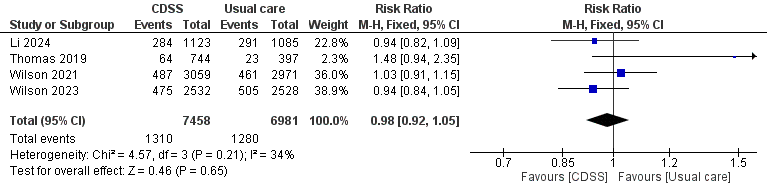


Figure S4: Forest plot of length of hospital stay, CI: confidence interval.


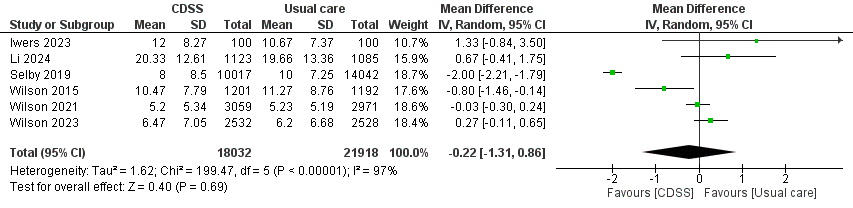


Figure S5: Forest plot of hospital cost, CI: confidence interval.


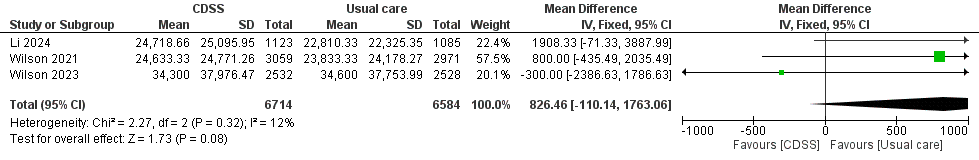


Figure S6: Forest plot of number of patients received fluids, CI: confidence interval.


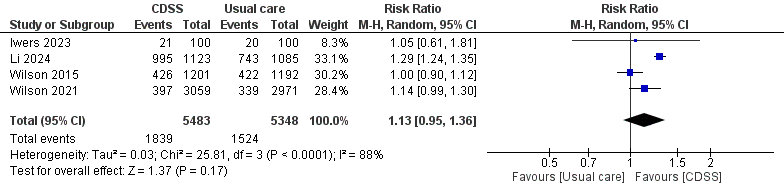


Figure S7: Forest plot of number of patients underwent renal ultrasonography, CI: confidence interval.


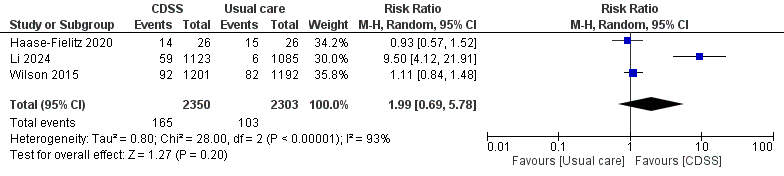


Figure S8: Forest plot of nephrologist consultation, CI: confidence interval.


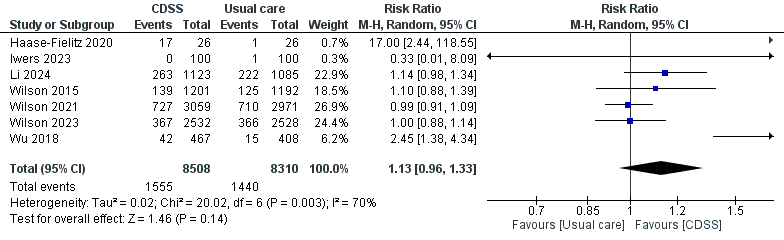

Supplement: Supplementary material.docx [file IRNF_A_2400552_SM5209.docx]
